# Intestinal Parasites

**Published:** 1906-09-15

**Authors:** J. M. Fortescue-Brickdale


### Hospital Clinics.

### INTESTINAL PARASITES. By J. M. Fortescue-Brickdale, M.A., M.D.Oxon.

### Ascaris Lumbricoides.

For some unexplained reason the diagnosis of intestinal worms is a very popular one with mothers in certain classes of society. We all know the small, thin child who is brought to the doctor because it picks its nose and is therefore thought to have worms; and we all know, too, that malt and cod-liver oil or some Syrupus ferri phosph. co. will improve the symptoms, though not exactly anthelmintics. Still, there is no doubt that many curious conditions are occasionally set up by worms. Recently some cases have been reported in which ascaris lumbricoides was more than suspected of setting up typical attacks of appendicitis. It is true that the ascaris was never actually found *in flagrante delicto*, but the worm was passed so soon after the attack that there was a strong *prima facie* case against it. The ascaris is said to have a special tendency to insert itself in small apertures; but though this is one of the statements which appears in all text-books of medicine, it is difficult to understand how evidence on the point was collected.

### Tœnidæ.

The most troublesome worm, from the point of view of treatment, is the tape-worm, owing to the frequency with which the head is left behind in the intestine, and grows fresh segments later on. Fowler has recently described a method of treatment which he has found very successful; it can be easily applied to most private patients, though his statistics are quoted from the Middlesex Hospital records, where these cases are usually admitted instead of being treated as out-patients. The patient is kept in bed for three or four days on a very small amount of food, two pints of beef-tea, a tin of Mason's essence, two rusks, and four ounces of port wine being all that is allowed. Two grains of cascara are given thrice daily. The duration of this treatment depends on the strength of the patient, but usually on the fourth day haustus sennæ co. ℥j. is given at 5 a.m., and at 9 a.m. 15 minims of extract of male fern is given in a capsule, and the dose repeated four times, at intervals of a quarter of an hour. At 11 a.m. the black draught is repeated. If the head has not been passed by 1 p.m. the male fern and black draught are again given as before, and, if necessary, a third course may be given; but after this the patient should be fed for a day, and the treatment begun afresh. The head of the worm should be sought for carefully, the vessel into which the motion is passed being covered with black crêpe, against which the fine head is more easily seen. This method is based on sound principles, and in practice has been found very efficacious. It is certainly worth the trouble of a few days in bed.

### Ankylostomiasis.

A species of worm at present rare in this country is the ankylostoma. It has, however, been found frequently among certain classes of miners in Cornwall, and the experience of the United States should put us on our guard against the possible invasion of our country by this parasite from abroad. The soldiers of the United States Army became infected in the Philippines, and returned home to distribute the unwelcome guest among the peaceful citizens of the States. It is as well, then, that medical men should keep an eye on recent methods of treatment of this condition, as it is possible that the knowledge might at any time become extremely useful. An interesting point in the prophylaxis is the value of common salt in destroying the young larvæ before they become encapsuled. This was experimentally demonstrated by Haldane and Boycott, and is further shown by the fact that salt mines and mines under the sea into which salt water can percolate are remarkably free from ankylostoma. The ordinary treatment by thymol has certain disadvantages. Apart from the fact that it is not uniformly successful, it may cause extreme cardiac depression and even death. As an alternative, Phillips has recently employed the method devised by Hermann, with encouraging results. The method is as follows: At 6 p.m. the patient takes a saline purge, and then no further food is allowed. The next morning, at 7 p.m., he takes a mixture containing eucalyptus oil, ♏ xv. (1 gram); chloroform, ♏ xv. (1.5 gram); castor oil, ad ʒvj. (20 grams). The dose is repeated in half an hour. In feeble, anæmic patients and in children smaller doses are given, or the same total in three doses. Should any cardiac weakness manifest itself after the first dose, the subsequent ones may be omitted. The patient is kept in bed until the bowels have acted, and is allowed no food. The method is said to be remarkably successful, and free from unpleasant by-effects. It may also be used for tape-worms.

